# Analysis of Photosynthetic Characteristics and UV-B Absorbing Compounds in Mung Bean Using UV-B and Red LED Radiation

**DOI:** 10.1155/2014/378242

**Published:** 2014-02-12

**Authors:** Fang-Min Li, Zhi-Guo Lu, Ming Yue

**Affiliations:** ^1^Key Laboratory of Resource Biology and Biotechnology in Western China, Ministry of Education, The School of Life Science, Northwest University, Xi'an, Shaanxi 710069, China; ^2^The College of Physics, Northwest University, Xi'an, Shaanxi 710069, China

## Abstract

Mung bean has been reported to have antioxidant, antidiabetic, anti-inflammatory, and antitumor activities. Various factors have important effects on the types and contents of plant chemical components. In order to study quality of mung bean from different light sources, mung bean seedlings were exposed to red light-emitting diodes (LEDs) and ultraviolet-B (UV-B). Changes in the growth parameters, photosynthetic characteristics, the concentrations of chlorophyll a and chlorophyll b and the content of UV-B absorbing compounds were measured. The results showed that photosynthetic characteristics and chlorophyll a and chlorophyll b concentrations were enhanced by red LEDs. The concentrations of UV-B absorbing compounds were enhanced by UV-B on the 20th day, while photosynthetic characteristics, plant length, and the concentrations of chlorophyll a and chlorophyll b were reduced by UV-B on the 40th day; at the same time the values of the stem diameter, plant fresh weight, dry weight, and the concentrations of UV-B absorbing compounds were enhanced. It is suggested that red LEDs promote the elongation of plant root growth and photosynthetic characteristics, while UV-B promotes horizontal growth of stems and the synthesis of UV-B absorbing compounds.

## 1. Introduction

Mung bean (*Phaseolus radiatus* L.) is a leguminous species grown in different parts of the world, primarily especially in Asia including China, India, Burma, and Thailand. Mung bean commonly is a common source of protein in the Asian diet or nutrient supplements [1]. Mung bean has been reported to possess antioxidant, antidiabetic, anti-inflammatory, antitumor and antimelanocytes, and antiangiotensin I-converting enzyme activities [2–8]. Mung bean contains free phenolic acids, bound phenolic acids, total phenolic, and anthocyanin. Correlation analyses between bioactivities and phytochemicals demonstrated that antioxidant bioactivity may be mainly contributed to phenolic compounds, whereas anthocyanins play an important role in the antidiabetic bioactivities [3]. Other reports showed that flavonoids including vitexin and isovitexin were the dominant components in mung bean [2, 9] and the content of vitexin was much higher than that of isovitexin in ethanol extracts [10]. It has been reported that mung bean has a strong antioxidant activity and isovitexin and vitexin contribute to most of the 1,1-diphenyl-2-picrylhydrazyl, ferric-reducing antioxidant power or 2,2′-azinobis-(3-ethylbenzthiazoline-6-sulphonate) radical scavenging ability [2].

Various factors including geographical location, climate change, temperature, and illumination time have important effects on the types and contents of plant chemical components, which are related to their bioactivity, functionality, and applications. Light quality is one of the most important factors in the regulation of plant growth, morphogenesis, photosynthesis, metabolism, and gene expression [11, 12]. For the photobiological research, ultraviolet-visible spectrum between 200 nm and 800 nm wavelength plays an important role in changes of chemical compounds of the organisms by irradiating them, especially compounds with ultraviolet (UV) absorption property [13, 14].

Compared to the ordinary fluorescent light source, the light-emitting diode (LED) light sources can provide a single wavelength of light quality with high photoelectric conversion efficiency, fixed wavelength, and low heat. LED light source is considered to be a new important light source in the field of plant physiology and plant cultivation. Previous studies indicated that the LED light sources were used in the research of photomorphogenesis [15], chlorophyll synthesis [16], and photosynthesis [17]. Recently the studies of this field attract more and more researchers focusing on the work [18, 19].

Many studies indicated that there were the practical problems of insufficient light intensity and limited spectral wavelength during the process of plant cultivation in the laboratory [20–22]. It is necessary to find effective ways to replace or assist the ordinary fluorescent light source, to improve research method for the plant, and to promote the plant quality. In this study, we used the red LEDs and ultraviolet-B (UV-B) radiation as additional light sources for the process of plant cultivation in the laboratory to determine the role of the different qualities of light source on growth and photosynthetic characteristics of mung bean.

## 2. Materials and Methods

### 2.1. Plant Materials

Mung bean (*Phaseolus radiatus *L. cv. Qindou 20) seeds were selected for uniform size. Mung bean seeds were obtained from Yangling Breeding Center of National Bean Engineering Research Center of China (Shaanxi, China).

### 2.2. Supplementary Light Treatments

The ordinary fluorescent light source (power 40 W) was purchased from Philips Inc. The light directly irradiated the seedling of mung bean from am 7:00 to pm 7:00 each day. The UV-B radiation was provided by filter Qin brand (Baoji Lamp Factroy, China) 30 W fluorescence sunlamps. They were filtered with 0.13 mm thick cellulose diacetate (transmission down to 290 nm) for UV-B radiation. The dose of UV-B irradiation was 0.861 kJ/m^2^ per day. The supplementary light treatments were shown in Table 1. The lamps were suspended above the plant at the height of 40 cm perpendicular to the ground.

Firstly, seeds were sterilized for 10 min by 0.1% HgCl_2_ and were grown in Petri dish (diameter 18 cm) after being washed for 50 min by flowing water. Until seeds were germinated, they were transplanted in basin (diameter 25 cm) which was filled with the ratio of peat : vermiculite : perlite for 3 : 1 : 1. One week after seed germination, the supplementary light treatments carried out seed germination. On the 20th day and 40th day of supplementary light treatments, organisms were sampled, respectively, for various analyses.

### 2.3. Effects Test

#### 2.3.1. Growth Parameter

The morphology including plant height, fresh weight, dry weight, root length, and stem diameter was measured. Mung bean seedlings were oven dried at 80°C until constant weight and being weighed using electronic scale as biomass (g).

#### 2.3.2. Photosynthetic Characteristics

Photosynthetic characteristics were measured with a photosynthesis meter (Photosynthesis Meter I-301, CID. Inc.). The water use efficiency was the ratio of photosynthesis and transpiration. The results of stomatal conductance, photosynthesis and water use efficiency were the mean values of the day.

#### 2.3.3. Determination of Chlorophyll a (chl a) and Chlorophyll (chl b) and UV-B Absorbing Compounds

The method for the measure of the concentration of chl a and chl b was extracted by acetone and determined following the reported methods [23]. Intact leaf samples of seedlings (fresh weight 0.5 g), which were at 5-6 leaves stage of development, were placed in a mortar and followed by the addition of silica of 0.2 g, CaCO_3_ of 0.2 g, and 15 mL 80% acetone. After thorough grinding, the samples were filtrated with two layers of filter paper by pump air and fixed to 25 mL with 80% acetone, and then the absorbance at 663 and 645 nm was determined, respectively. Chlorophyll concentration was calculated and expressed as mg/g FW.

Fresh samples of 0.5 g were taken from the epicotyls and extracted in 10 mL acidified methanol (methanol-water-hydrochloric acid, 79 : 20 : 1, v/v) for UV-B absorbing compounds, according to the procedure of Mirecki and Teramura [24]. The hydrochloric acid was 36% HCl. Extract absorbance at 300 nm was measured with a spectrophotometer (UV-2100; Shimadzu, Columbia, MD, USA) and the absorbance was arbitrarily used for analysis.

### 2.4. Statistical Analysis

All experiments were performed in six times repeatedly. Statistical analyses were performed with SPSS 11.5 for windows. The results were expressed as the means ± standard error (SE) of triplicate. The data were subjected to one-way analysis of variance (ANOVA) and the significance of difference between samples means was calculated by Duncans' multiple range test and *P* values less than 0.05 were considered significant.

## 3. Results

### 3.1. Growth Parameters

It was observed that the values of the growth parameters of mung bean seedlings were irradiated for 20th day by red LEDs and UV-B was not significantly different compared with that of the ordinary fluorescent light (Table 2). However, the red LEDs treatment caused a significant increase (*P* < 0.05) of the values of plant height, fresh weight, dry weight, and root length compared with that of the ordinary fluorescent light for 40th day. With the UV-B radiation for 40th day, an obvious decrease (*P* < 0.05) of plant height was observed, while it induced a marked increase (*P* < 0.05) in fresh weight, dry weight, and stem diameter.

### 3.2. Photosynthetic Characteristics

The red LEDs treatment induced a significant increase (*P* < 0.05) in the values of the photosynthetic characteristics for the two durations (Table 3). However, a significant decrease (*P* < 0.05) was observed in the values of the photosynthetic characteristics of the UV-B radiation for 40th day.

### 3.3. Determination of chl a and chl b

**Figure 1:**
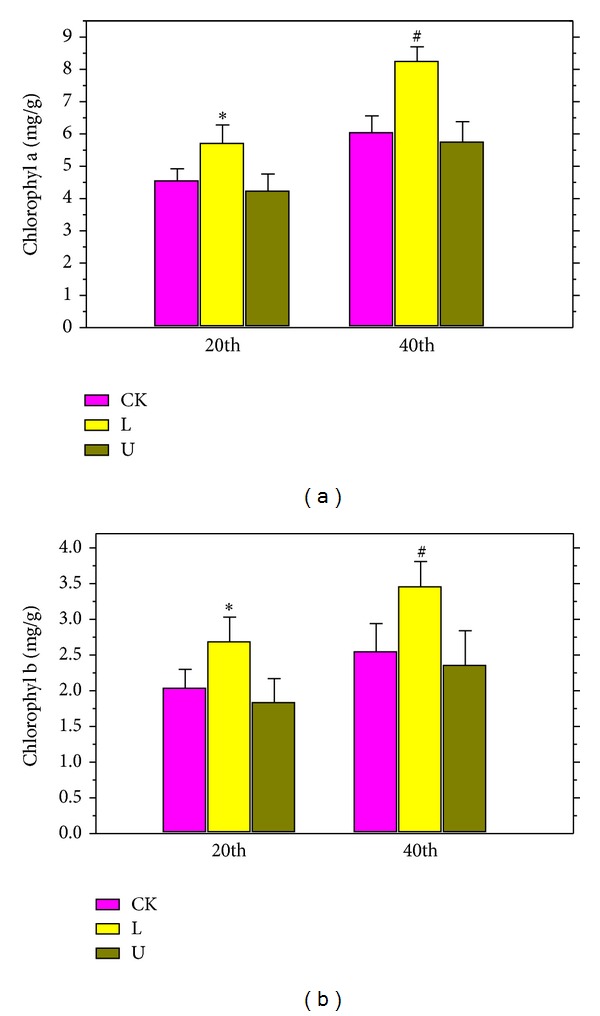
Effects of red LED and UV-B on the chl a (a) and chl b (b) of the mung bean. CK in figure refers to use ordinary fluorescent light as treat light sources. L in the figure refers to using both ordinary fluorescent light and red LEDs. U in the figure refers to using both ordinary fluorescent light and UV-B radiation light. Data are means and SE of six replicate plants. Error bars represent standard errors. Means with different letters above bars were significantly different at the *P* < 0.05 level (*n* = 6) according to Duncan's multiple range test (*P < 0.05; ^#^ *P* < 0.05).

The concentrations of chl a and chl b may affect the values of the photosynthetic characteristics to a certain extent. It was obvious that the red LEDs treatment induced statistically significant increases (*P* < 0.05) not only in the values of the photosynthetic characteristics (Table 3) but also in the concentrations of chl a and chl b (Figure 1) for the two durations. Compared with the ordinary fluorescent light, the UV-B radiation did not cause significant differences.

### 3.4. Determination of UV-B Absorbing Compounds

**Figure 2:**
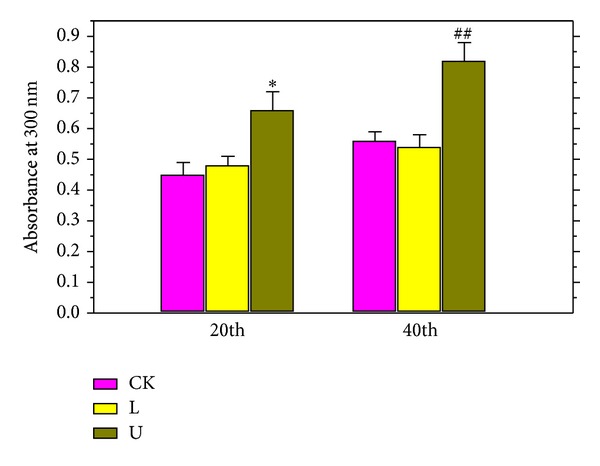
Effects of red LED and UV-B on the UV-B absorbing compounds of the mung bean. CK in the figure refers to use ordinary fluorescent light as treat light sources. L in Figure refers to the use of both ordinary fluorescent light and red LEDs. U in the figure refers to using both ordinary fluorescent light and UV-B radiation light. Data are means and SE of six replicate plants. Error bars represent standard errors. Means with different letters above bars were significantly different at the *P* < 0.05 level (*n* = 6) according to Duncan's multiple range test (*P < 0.05; ^##^ *P* < 0.01).

With the treatment of UV-B radiation, the concentrations of UV-absorbing compounds were increased dramatically and the same trend of results was shown in Figure 2. UV-B radiation-treated seedlings resulted in a notably increase in the concentrations of UV-absorbing compounds for the two durations (*P* < 0.05). However, red LEDs did not cause significant difference in comparison to the ordinary fluorescent light.

## 4. Discussion

LEDs are a promising irradiation source for plant growth in space for long life, minimal mass, volume, and being a solid state device. The red LEDs (wavelength 650 nm) were used as a supplementary light source for the greenhouse tomato in 1982, which was reported earlier by Japan's Mitsubishi Corporation [25]. During the process of laboratory cultivation, it has been reported that the ordinary fluorescent light lacked the ultraviolet part of the solar spectrum background, which was essential growth factor to play an important biological role [20, 26].

However, it was difficult to use a mixed-use LED light sources during the process of plant cultivation completely [27]. Therefore, we used red LED and UV-B as supplementary light sources for the ordinary fluorescent light to study the role of these light sources in plant cultivation. Our results showed that these light sources were obviously increased for growth and photosynthetic characteristics of mung bean. The red LED light source promoted the growth of the mung bean root (Table 2), which was useful to absorb the nutrients and water of the soil.

Chlorophyll concentrated in the chloroplast grana is the main pigment to capture the energy for photosynthesis in green plants. The results showed that the red LED light source can significantly increase the concentrations of the chlorophyll (Figure 1), which effectively promoted the photosynthesis and water use efficiency (Table 3). On the contrary, UV-B radiation induced a notably decrease in the photosynthesis and water use efficiency. It was suggested that the UV-B treatment will reduce the stomatal opening degree of mung bean and then affect the gas exchange in photosynthesis.

UV absorption compounds in leaves are an important class of pigments including flavonoid, flavonol, cinnamon, and anthocyanin, which determine the color changes of many plants and are very sensitive to light. They play an important role in protective effect as a class of secondary metabolites [28, 29], which are related to antioxidant, antidiabetic, anti-inflammatory, antitumor and antimelanocytes and antiangiotensin I-converting enzyme activities [2–8]. The previous studies have reported that vitexin and isovitexin were major flavonoid in the ethanol extract of mung bean and vitexin content was much higher than isovitexin in ethanol extracts from mung bean sprout [3, 9]. However, another report showed that no significant difference in levels of vitexin and isovitexin was observed in mung bean sprout of the same cultivar tested in the experiments [2]. Since UV absorption compounds have an absorption peak in UV-B radiation scope, it could be found that the UV-B treatment caused a significant increase in UV absorption compounds (Figure 2). Compared with the ordinary fluorescent light, red LEDs did not induce significant differences in UV absorption compounds. Red LED and UV-B as supplementary light sources have an important effect on plant growth and chemical components.

## Figures and Tables

**Table 1:** The treatments of the experiment.

Treatment	Ordinary fluorescent light	Red LEDs	UV-B radiation
CK	Ordinary fluorescent light	/	/
L	Ordinary fluorescent light	Red LEDs	/

U	Ordinary fluorescent light	/	UV-B radiation

The lamps were suspended above the plant at the height of 40 cm perpendicular to the ground.

**Table 2:** Effects of red LEDs and UV-B on the growth parameter of the mung bean.

Duration	Treatment	Fresh weight	Dry weight	Plant height	Root length	Stem diameter
		g/seedling	g/seedling	cm/seedling	cm/seedling	mm/seedling
20 d	CK	220.3	55.6	28.2 ± 2.1	6.38 ± 0.64	4.69 ± 0.45
	L	231.4	57.3	29.4 ± 2.6	6.59 ± 0.42	5.03 ± 0.61
	U	228.8	54.9	27.3 ± 2.4	6.08 ± 0.55	5.21 ± 0.36

40 d	CK	373.6	89.2	43.7 ± 2.7	7.69 ± 0.68	6.39 ± 0.74
	L	491.8^#^	110.6^#^	44.5 ± 3.0	9.04 ± 0.77^#^	6.65 ± 0.43
	U	460.7^#^	108.5^#^	38.2 ± 3.5^#^	7.32 ± 0.48	7.69 ± 0.63^#^

Note: different letters followed the data of same index at the same treatment time indicate significant difference among treatments. Means with pound sign (^#^) were significantly different at the *P* < 0.05 level (*n* = 6) according to Duncan's multiple range test (^#^ *P* < 0.05).

**Table 3:** Effects of red LEDs and UV-B on the photosynthetic characteristics of the mung bean.

Duration	Treatment	Photosynthesis	Stomatal conductance	Water use efficiency
		(*μ*mol/m^2^/s)	(mmol/m^2^/s)	WUE
20 d	CK	4.85	77.3	6.5
	L	5.62*	90.6*	9.8*
	U	4.63	75.9	7.0

40 d	CK	6.23	110.7	13.3
	L	7.49^#^	142.3^#^	19.6^#^
	U	5.33^#^	90.8^#^	9.1^#^

Note: different letters followed the data of same index at the same treatment time indicate significant difference among treatments. Means with asterisk (*) or pound sign (^#^) were significantly different at the *P* < 0.05 level (*n* = 6) according to Duncan's multiple range test (*P < 0.05; ^#^ *P* < 0.05).
